# Knowledge and Other Predictors of Child Welfare Clinic Completion among Children Aged 24–59 Months in the Garu-Tempane District of Northern Ghana: A Cross-Sectional Study of Caregivers

**DOI:** 10.1155/2020/6840609

**Published:** 2020-08-11

**Authors:** Maxwell Nibelo, Emmanuel Manu

**Affiliations:** ^1^University of Health and Allied Sciences, PMB 31, Hohoe, Ghana; ^2^Department of Population and Behavioural Sciences, University of Health and Allied Sciences, PMB 31, Hohoe, Ghana

## Abstract

**Background:**

While completion of the Child Welfare Clinic (CWC) schedule for children remains a crucial factor in the prevention of illness and promotion of better child health, there has been low attendance among caregivers in Ghana. This study examined knowledge of 220 caregivers of children aged 24–59 months on CWC and other factors influencing attendance in the Garu-Tempane District of Northern Ghana.

**Methods:**

This health facility-based descriptive cross-sectional study was carried out among caregivers of children using a structured questionnaire. Descriptive and inferential statistics comprising frequency, percentage, Fisher's exact test, and logistic regression were adopted in analysing the data.

**Results:**

Less than half (46.9%) of the children completed their CWC schedules. Meanwhile, caregivers' knowledge on CWC was 97.7%. Children aged 37–48 months (AOR = 0.42, 95%CI = 0.21–0.86, *p*=0.017) and 49–59 months (AOR = 0.27, 95%CI = 0.10–0.77, *p*=0.014), respectively, had lower odds of completing CWC. Children with caregivers not having any formal education also had lower odds of completing CWC (AOR = 0.45, 95%CI = 0.21–0.95, *p*=0.036).

**Conclusion:**

Educational programmes on the importance of CWC completion should focus on caregivers with children aged 37 months and above and those caregivers with low educational level. It is further recommended that studies be conducted to explore the extent of association between caregivers' marital status, occupation, level of knowledge, and child CWC completion in the Garu-Tempane District.

## 1. Introduction

Child Welfare Clinics (CWCs) are a critical component of child healthcare in the developing world as they serve as a platform for health workers to interact with caregivers and build consensus based on improved and practicable ways of promoting optimal child growth [1, 2]. For instance, growth monitoring, vaccination against preventable and infectious diseases, and health education for caregivers, as well as other health promotional services are provided at these CWCs [3]. Although, in Ghana, child immunization is completed at 24 months of age, other essential services such as growth monitoring, deworming, screening for malnourished children, and management and referral cases are undertaken at CWC sessions until a child reaches 60 months of age [4]; hence, there is need for a child to attend CWC until such age. CWC attendance, however, usually declines from 24 months in the country when all immunizations have been completed [5].

The Upper East region of Ghana is one of the regions with the worst CWC attendance for children aged 24 months and above [6]. For instance, in the Garu-Tempane District, the CWC attendance for children aged 24–59 months declined from 20.4% in 2016 to 20% in 2017 and further declined to 19% in 2018 [7]. Hence, the phenomenon has been of concern for the Ghana Health Service [6] as it strives to improve health outcomes of children under five years of age, of which scheduled CWC attendance is one of the key strategies in achieving that [8]. Non-completion of CWC among children has, meanwhile, been attributed to the lack of knowledge on the importance of CWC, especially post-immunization completion among caregivers of children [9], and is often the case in most developing countries, including Ghana [10].

**Figure 1:**
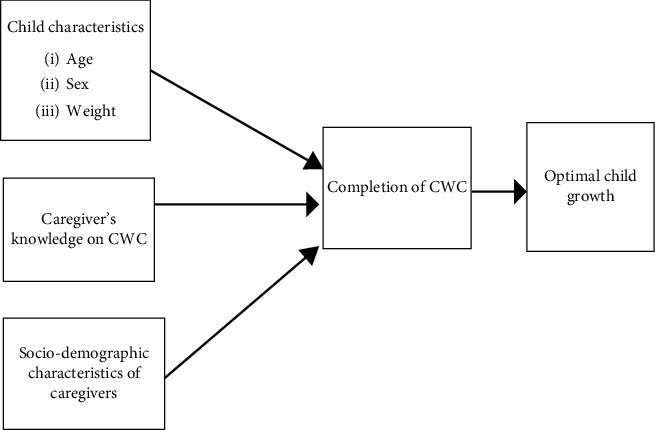
Conceptual framework of the child welfare clinic attendance.

However, studies conducted on the subject in Ghana either looked at knowledge of caregivers on the CWC schedule or reasons for non-completion of CWC and, thus, failed to ascertain the determinants of CWC completion [5] to inform CWC-related education. Recently, some studies have ascertained the reasons behind low CWC attendance, as well as the attitude and practices of mothers towards CWC in the country [9, 10]. However, all these studies [5, 9, 10] focused on CWC completion among children in Southern Ghana, a part of the country which is economically and socially advantaged compared to the northern sector of the country [11–13]. One of the few and current studies conducted in the Mumprusi District of Northern Ghana also only focused on growth monitoring and promotion practices among health workers [14], neglecting caregivers, who are essential in determining child CWC completion. The current study, therefore, plugs a literature gap by examining knowledge of caregivers of children aged 24–59 months and other predictors of CWC completion in the Garu-Tempane District of Northern Ghana, based on the tenets of a conceptual framework adapted from the work of Mahama [15], as depicted in Figure 1.

Source: adapted from the work of Mahama [15].

## 2. Materials and Methods

### 2.1. Study Setting

The study was conducted in the Garu-Tempane District of the Upper East Region of Northern Ghana from May to August, 2019. The district is one of the 13 administrative districts and municipalities in the Region. The district shares boundaries with the Republic of Togo to the east and Bawku West and Binduri districts to the west, with Bunkpurugu Yunyoo District to the south and the Bawku Municipal in the north [7]. The district is the largest in land mass and the second in population in the region and covers an area of 1,230 square kilometres. It is predominantly rural (95.6%) with an estimated population of 154, 214. The predominant ethnic groups in the district are the Kusasis, Busangas, Mosis, Bimobas, and the Mamprusis [7]. The district has 56 health facilities made up of 11 health centres, 5 private clinics, and 42 operational CHPS Zones. CWC services are rendered at facility-based (static) and outreach clinics in communities on a weekly basis [7]. The district was chosen for the study due to its low rate of CWC attendance among children aged 24 to 59 months [7].

### 2.2. Study Design

This was a descriptive facility-based cross-sectional study which was conducted among caregivers of children 24–59 months of age in the Garu-Tempane District of Northern Ghana. The descriptive design was guided by the positivist research philosophy which underpinned the fact that the present study was conducted quantitatively [16]. A paper-based questionnaire was developed to solicit responses from the caregivers. The questionnaire consisted of three main sections: socio-demographic, knowledge on CWC, and CWC completion sections. Knowledge variables that were assessed include understanding of growth monitoring, required monthly CWC sessions, the importance of CWC attendance, and the benefits of CWC attendance after completion of the immunization schedule. The questionnaire was pretested among twenty respondents in three facilities from a neighbouring district that was not part of the study to determine its strengths and weaknesses concerning its reliability and validity. Reliability of the questions in assessing knowledge on CWC was determined by subjecting them to Cronbach's alpha test which yielded an acceptable reliability coefficient of 0.646 as per van Griethuijsen et al.'s [17] interpretation. Knowledge was assessed on the meaning, components and frequency of CWC, the importance of regular CWC attendance, importance and reasons for CWC continuation after completion of the immunization schedule, and on the uses and importance of the growth chart. Twenty expected correct responses from twenty questions (Table 1) were used to measure the overall level of knowledge of respondents on CWC with an average mark of ten (10). An individual who scored up to the average mark or above was classified to have a good level of knowledge on CWC, while a score below the average mark was considered a poor knowledge on CWC. Hence, respondents who scored up to the average score or above were classified as having good knowledge on CWC whiles those who scored below the average score were considered to have poor knowledge on CWC [18]. The proportion of children who completed CWC attendance by age was determined by inspecting their child health record booklets.

### 2.3. Source Population

The source population consisted of all caregivers with children aged 24 to 59 months in the Garu-Tempane District of Northern Ghana.

### 2.4. Study Population

The study population consisted of caregivers of children aged 24 to 59 months in the Garu-Tempane District of the Upper East Region of Ghana who had resided in the region for a minimum of one year and were having CWC cards for their children.

### 2.5. Sample Size Determination and Sampling

The sample size for this study was determined using Cochran's [19] formula *n*=*z*^2*∗*^*p*(1 − *p*)/*d*^2^ based on the prevalence (*p*) of incomplete CWC attendance of 93.4% (0.934) among caregivers with children aged 24 to 59 months in the Assin North District of Ghana [5], with *z* = 1.96 and *d* = 0.025 at a 95% confidence level. Substituting the values into the formula yielded a sample size of 194. However, data were collected from 220 participants, of which 213 were used in the analysis due to inconsistencies detected in 7 questionnaires during the data cleaning process.

A multistage sampling procedure was used to select individual caregivers. At the first stage, the district was stratified into the existing nine sub-districts. The second stage involved the selection of two CWC outreach sites from each stratum using a simple random sampling technique. This was performed by writing all the names of the outreach sites in each sub-district on paper and putting them in a container. The containers were, then, thoroughly shaken, and two papers were drawn at random without replacement. Thirdly, lists of caregivers with children between 24–59 months attending CWC at the selected outreach sites were obtained from the sub-districts. The sample size was proportionately allocated to each outreach site based on the total number of children within 24–59 months attending CWC at each site, based on the required sample size for the study. Lastly, in each outreach site, caregivers with children within 24–59 months were identified and selected using simple random sampling (YES or NO) after they consented to participate in the study. Depending on the attendance of that day, the “YES” corresponded to the proportion of sample size allocated to the outreach site with the rest being “NO”. All the papers were put in a bowl and thoroughly mixed together. All caregivers who came for CWC and met the inclusion criteria, then, balloted by picking a piece of paper from the bowl. Any caregiver who picked YES was, then, interviewed. CWCs are conducted once every week across various outreach CWC centres in the district [7]. Data collection was, therefore, performed on every CWC day in each selected centre, thus once a week, until the required sample size was reached.

### 2.6. Data Collection Procedure

Data were collected using a pretested semistructured questionnaire after obtaining ethical clearance from the University of Health and Allied Sciences' Research Ethics Committee. Two research assistants from the School of Public Health, University of Health, and Allied Sciences were trained as field assistants for two days on the data collection tool. The data collectors were informed on the purpose of the study and taken through the data collection tool. This was to ensure that data collectors understood how to ask questions appropriately to enable the respondents to provide appropriate answers. Data were collected on the socio-demographic characteristics of caregivers, child characteristics, and caregivers' knowledge on CWC and child completion of CWC. Data were collected at the selected CWC centres on every CWC day, once a week, until the required sample size was obtained. The data collectors were supervised on the field by both authors. All interviews were conducted face-to-face.

### 2.7. Study Variables

Explanatory variables measured in this study were the age of the child, sex of the child, birth weight of the child, age of the caregiver, sex of the caregiver, the caregiver's relationship with the child, marital status of the caregiver, type of marriage, occupation of the caregiver, level of education of the caregiver, religion of the caregiver, and tribe of the caregiver. The outcome was CWC completion at the current age.

### 2.8. Operational Definitions

#### 2.8.1. Good Knowledge

Study participants who answered at least half of the knowledge questions correctly.

#### 2.8.2. Poor Knowledge

Study participants who answered less than half of the knowledge questions correctly.

#### 2.8.3. Completion of CWC

Children of study participants who were able to attend the required number of CWC sessions at their current age.

#### 2.8.4. Non-completion of CWC

Children of study participants who had missed a scheduled CWC session at their current age.

### 2.9. Data Analysis

Data were coded and entered into EpiData version 4.2.0 and exported to SPSS Version 21.0 for analysis. Exploratory data analysis was performed to check missing values. Fisher's exact tests were conducted to determine the relationship between the explanatory variables and the outcome variable (completion of CWC). Two binary logistic regression models (crude and adjusted) were, then, used to determine the strength of the association between the explanatory variables and completion of CWC. All statistical analyses were considered significant at *p* < 0.05. The results are presented in tables and graphs.

### 2.10. Ethical Issues

Ethical approval was obtained from the University of Health and Allied Sciences (UHAS) Research Ethics Committee (UHAS-REC) (UHAS-REC.A.8 [32] 18-19). Permission was also obtained from the Upper East Regional Health Directorate and the Garu-Tempane District Health Directorate, as well as the health facilities where data were collected. Written informed consent was obtained from the respondents. Confidentiality and anonymity for respondents and the CWC facilities were also ensured by not reporting names and other personal identifiers.

## 3. Results

### 3.1. Socio-Demographic Characteristics of Respondents

Table 2 presents the socio-demographic characteristics of caregivers and their children. A total of 213 complete questionnaires out of the 220 caregivers interviewed were used in the analyses. The majority (56.8% (121/213)) of the children were between ages of 24 and 36 months. Most of the children were males (51.6% (110/213)) and with birth-weights between 2.5 and 4.0 kg (77.5% (165/213)). Ninety-five percent (203/213) of the caregivers were females and 46.5% (99/213) of them were between the ages of 20 and 29 years. A greater proportion (91.5% (195/213)) of the caregivers was parents of the children. Also, the majority (97.7% (208/213)) of the caregivers were married and in monogamous marriage (62.4% (133/213)). Most (52.1% (111/213)) of the caregivers never had any formal education, and a majority (71.8% (153/213)) of them were farmers, with Kusaasi being the dominant tribe (59% (126/213)).

### 3.2. Knowledge of Caregivers on Child Welfare Clinic

The level of knowledge of caregivers was measured using the questions presented in Table 1. Majority (97.7% (208/213)) of caregivers knew that the meaning of growth monitoring is weighing. Less than half 49.3% (105/213) knew that immunization is not part of growth monitoring activities, with the majority (96.7% (206/213)) knowing that CWC sessions are attended once a month. The most (86.4% (184/213)) agreed that regular CWC attendance is important for growth monitoring. However, only 35.2% (75) knew that regular CWC attendance is important for correction of growth falter in a child. Majority 60.6% (129/213) knew that regular CWC attendance is important for monitoring the child's health. Majority (57.7 (122/213)) knew that regular CWC attendance is not to determine if the child is sick. Over half 57.7 (123/213) of the respondents knew that regular CWC attendance is important for the prevention of diseases. All (100% (213/213)) respondents agreed that regular CWC attendance is important. Most respondents (96.2% (205/213)) knew that it is important to attend CWC after completion of the immunization schedule and also knew that child growth and monitoring were one of the reasons for CWC continuation after completion of immunization schedules (85.5% (182/213)). However, few (43.7% (93/213)) of the respondents knew that seeking health and nutritional advice is one of the reasons for CWC continuation, after completion of the immunization schedule. The majority (65.3% (139/213)) correctly knew that continuation of CWC was not just to comply with a government's policy or to comply with a health worker's directive (57.7% (123/213)). The majority of the respondents (80.3% (171/213)) knew that the growth chart is used to monitor a child's health, indicates how a child is growing (68.5% (146/213)), does not indicate the direction child growth (53.5 (114/213)), and not for education of mothers on growth monitoring (72.8% (155)). However, only 40.4% (86/213) knew that the chart is not meant to help caregivers to care for a child. Overall, a majority (97.7% (208/213)) of respondents had good knowledge on CWC.

### 3.3. Completion of the CWC Schedule among Children Aged 24–59 Months

**Figure 2:**
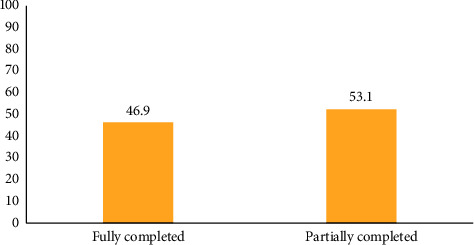
Completion of CWC at the current age (*n* = 213).

Figure 2 presents child welfare clinic schedule completion among children aged 24–59 months. Less than half (46.9% (100/213)) of the children had completed their CWC sessions at their current age at the time of the study.

### 3.4. Predictors of Child Welfare Clinic Completion among Children Aged 24–59 Months

From Table 3, Fisher's exact tests conducted revealed that the age of the child (*p*=0.002), marital status of the caregiver (*p*=0.022), occupation of the caregiver (*p*=0.003), level of education of the caregiver (*p* ≤ 0.001), and knowledge of the caregiver on CWC (*p* ≤ 0.001) were significantly associated with CWC completion. Logistic regression analyses conducted revealed that children aged 37–48 months (AOR = 0.42, 95%CI = 0.21–0.86, *p*=0.017) and 49–59 months (AOR = 0.27, 95%CI = 0.10–0.77, *p*=0.014), respectively, had lower odds of completing CWC. Children with caregivers not having any formal education also had lower odds of completing CWC (AOR = 0.45, 95%CI = 0.21–0.95, *p*=0.036).

## 4. Discussion

This study examined knowledge and other predictors of CWC completion among caregivers of children aged 24 to 59 months in the Garu-Tempane District of Northern Ghana. We found that most respondents had good knowledge on CWC. However, compared to previous studies conducted in the country, our finding showed a significant improvement in the knowledge level on CWC among caregivers. For instance, Senkyire [10] found poor knowledge level on vaccines and vaccine-related issues associated with CWC among caregivers in Madina, Accra. The improvement in the knowledge level on CWC found in the present study could be as a result of improvement in the quality of education that caregivers receive from health workers during CWC sessions. According to Nsiah-Asamoah et al. [20], health education given to caregivers during CWC sessions in rural parts of Ghana, such as the Garu-Tempane District, often focuses on the importance of attending CWC and vaccination of children. Thus, such education programmes targeting caregivers are well understood by the target audience. Moreover, several non-governmental organizations are working in the northern parts of the country to improve child and maternal health outcomes by constantly educating caregivers on the importance of CWC [21]. Also, compared to other studies conducted on the continent, such as in South Africa, our finding on the knowledge level on CWC was higher. A study conducted by Blaauw et al. [22] found a relatively lower (79.2%) knowledge level on the importance of CWC among South African mothers. This means that education on the importance of CWC completion in our study district is at par with similar programmes on the continent, if not better.

Although a relatively good knowledge on CWC was found among caregivers, it did not translate into CWC completion among children. We found that only 46.9% of the children in our study fully attended the required CWC sessions as per their current age. Although CWC attendance was known to be on the decline for children aged 24–59 months in the Garu-Tempane District, this had not been empirically ascertained. Our finding, therefore, fills a knowledge void by empirically ascertaining the CWC completion rate for children aged 24 to 59 months in the district. The low level of CWC completion among children found in the current study was, however, not in consonance with the results of a study conducted by Kazungu and Adetifa [23] that found an appreciable level (78.8%) of Ghanaian children who completed their required CWC sessions at their current age. The difference in CWC completion rate between our study and that of Kazungu and colleagues could, however, be due to the fact that while the current study focused on only one district in Northern Ghana, which is an impoverished geographical area, the latter aggregated data on the entire country which does not give a true reflection of happenings in individual districts. Hence, the impression created that Ghana has a good CWC completion rate as reported in other studies should be interpreted with caution.

With reference to factors that influenced CWC completion, our logistic regression model showed that the child's age and caregivers' educational level were associated with child CWC completion. Children aged 37 months and above were found to be less likely to complete CWC sessions compared to children aged 36 months and below. A similar finding has been reported in Ghana in the past. A study conducted in the Assin North District found that CWC attendance declines as children grow older [5]. On the African continent, similar findings have also been reported. A study conducted by Nyabuti [24] in Nyamira County, Kenya, found that CWC attendance declined as children grow. This implies that the challenge of non-CWC completion among older children is not peculiar to the Garu-Tempane District and Ghana in particular but a continental one. In explaining why older children do not complete CWC, Mahama [15] posit that many caregivers do not see the essence of sending their children to CWC after immunization schedules have been completed. This explanation might have accounted for our finding where children older than 36 months were less likely to complete their CWC sessions.

With reference to maternal factors, the marital status of caregivers was one of the factors found to be associated with a child's CWC completion. However, the extent of the association was not known as the multivariate regression analysis showed no statistical significance. Meanwhile, a preprint published by Konlan et al. [25] revealed that children of married caregivers were more likely to complete the CWC schedule, even after completion of the immunization schedule, in rural parts of Tamale, Northern Ghana, compared to children of unmarried caregivers. With our study participants sharing similar socio-geographical and economic characteristics as those of Konlan et al. [25], it could be assumed that children of married women in the Garu-Tempane District of Northern Ghana are more likely to attend and complete the CWC schedule, even after completion of the immunization schedule. Our finding, therefore, adds to build knowledge on the role of marriage in child health growth promotion and monitoring in Northern Ghana. This finding, though unverified, could mean that married women in our study district are supported by their husbands as far as child growth monitoring and promotion activities such as CWC attendance are concerned. It has been established that, in situations where married women do not receive husband's support, they do not regularly attend child growth monitoring and promotion activities [26].

Another caregiver-related factor that had an influence on a child's CWC completion was the occupation of a caregiver. Although the association was not significant after other variables were controlled for, children of caregivers who were farmers were less likely to complete CWC sessions compared to children of government workers. This may be due to the fact that CWC sessions are normally organized in the morning [9], and since farmers need to be in their farms early in the morning, taking into consideration the high day-time temperatures in the northern part of Ghana [27], they fail to attend. It, therefore, becomes a matter of preference for caregivers, and considering the poor socio-economic conditions in the northern part of Ghana [28], especially for peasant farmers [29], caregivers who are subsistent farmers cannot afford to miss out on their farming activities as that is their source of livelihood. Moreover, the finding could be explained by the interaction between education, knowledge, and completion of CWC. Government workers in Ghana are often more educated than farmers [30]; hence, the knowledge level on the importance of CWC completion among public and civil servants could be subsequently higher than that of farmers. The implication of this finding is that CWC sessions should take into consideration the timing during which such sessions are conducted. For instance, conducting CWCs in the evening or on days when communities do not indulge in farming activities could optimize the utilization of CWC services.

Moreover, children of caregivers who had no formal education were less likely to complete all CWC sessions compared to children of caregivers who had primary education. This means that the educational level of caregivers influences the likelihood of child CWC completion. It is possible that caregivers of higher educational level have higher knowledge on the importance of CWC. Thus, our finding is in consonance with previous studies which found education as an important predictor of parental health decision making for children [31, 32]. This is so because caregivers with least formal education may have little or no knowledge on activities conducted at CWC after completion of the immunization schedule or may view such activities as irrelevant, as already established by other researchers [5, 33]. The implication is that educational messages on the importance of CWC completion in the district need to consider the educational levels of caregivers to ensure that targeted health education is provided instead of mass education.

Lastly, caregivers' knowledge on CWC was also found to be significantly associated with child CWC completion. Children of caregivers' with good knowledge on CWC were found to be more likely to complete CWC sessions in the unadjusted model. However, after other variables were controlled for, the association was insignificant. Plausible explanation to this finding could be that caregivers who have good knowledge on CWC are likely to know the benefits of CWC continuation and after immunization schedules are completed. Hence, they continue to attend CWC sessions even after the child's completion of immunization schedules [10]. Our finding, thus, points out to Engell et al.‘s [34] postulation that good knowledge of caregivers on CWC often translates into improved health and educational outcomes for children. Our finding, therefore, enriches the literature on the role the knowledge gap plays in influencing optimal child growth and development. Children whose caregivers have poor knowledge on CWC are more likely to suffer from benefits of CWC as a result of non-completion [9, 10], thereby affecting their growth and development. Hence, increasing knowledge on the importance of CWC completion for all children should be of topmost priority to health decision makers in the Garu-Tempane District.

## 5. Strengths and Limitations

The strength of our study is that CWC attendance cards of the sampled children were inspected to ascertain that their actual CWC attendance was recorded instead of relying on verbal reports of caregivers which could have led to recall bias. Also, caregivers were randomly selected from the district which ensured that there was heterogeneity among the study respondents, making it possible to generalize our findings. One of the limitations of the study is that we did not take into account the influence of caregivers' beliefs and societal and health system factors on CWC attendance and completion, which could have played major role in the non-completion of CWC schedules. Moreover, reasons for non-completion of CWC were not solicited from respondents.

## 6. Conclusions

Although knowledge of caregivers on CWC was high, child CWC completion rate was low and declined with increasing age. Moreover, children of caregivers with low educational attainment were less likely to complete CWC. It is, therefore, recommended that educational programmes on the importance of CWC completion should focus on caregivers with children aged 37 months and above and caregivers with low educational level. Also, further studies should be conducted to explore the extent of association between caregivers' marital status, occupation, and child CWC completion in the district. Such a study should also take into consideration caregivers' beliefs and societal and health system factors that influence CWC completion.

## Figures and Tables

**Table 1:** Knowledge of caregivers on CWC.

Item	Expected response	Given responses *n* = 213	*n* (%)
Knowledge on meaning, components, and frequency of growth monitoring and CWC			
Growth monitoring means weighing	Yes	Yes	208 (97.7)
		No	5 (2.3)
Immunization as part of growth monitoring activities	No	Yes	108 (50.7)
		No	105 (49.3)
Treatment of minor ailments as part of growth monitoring activities	No	Yes	46 (21.6)
		No	167 (78.4)
CWC sessions are attended once in a month	Yes	Yes	206 (96.7)
		No	7 (3.3)

Knowledge on the importance of regular CWC attendance			
Regular CWC attendance is important for growth monitoring	Yes	Yes	184 (86.4)
		No	29 (13.6)
Regular CWC attendance is important for the correction of growth falter in a child	Yes	Yes	75 (35.2)
		No	138 (64.8)
Regular CWC attendance is important for monitoring child health	Yes	Yes	129 (60.6)
		No	84 (39.4)
Regular CWC is important to determine if a child is sick	No	Yes	91 (42.7)
		No	122 (57.3)
Regular CWC attendance is important for prevention of diseases	Yes	Yes	123 (57.7)
		No	90 (42.3)
Regular CWC important	Important	Important	213 (100)
		Not important	0 (0)

Knowledge on the importance and reasons for CWC continuation after the completion of the immunization schedule			
CWC is important after immunization schedules are completed	Important	Important	205 (96.2)
		Not important	5 (2.4)
		Do not know	3 (1.4)
Child growth monitoring as a reason for CWC continuation		Yes	182 (85.5)
	Yes	No	31 (14.5)
Seeking of health and nutritional advice as a reason for CWC continuation	Yes	Yes	93 (43.7)
		No	120 (56.3)
Complying with the government's policy as a reason for CWC continuation	No	Yes	74 (34.7)
		No	139 (65.3)
Complying with health workers' directive as a reason for CWC continuation	No	Yes	90 (42.3)
		No	123 (57.7)

Knowledge on the uses and importance of the growth chart			
The growth chart is used to monitor a child's growth	Yes	Yes	171 (80.3)
		No	42 (19.7)
The growth chart indicates how a child is growing	Yes	Yes	146 (68.5)
		No	67 (31.5)
The growth chart indicates the direction a child should grow in	No	Yes	99 (46.5)
		No	114 (53.5)
The growth chart is for the education of mothers on growth monitoring	No	Yes	58 (27.2)
		No	155 (72.8)
The growth monitoring chart is to help caregivers care for the child	No	Yes	127 (59.6)
		No	86 (40.4)
Overall knowledge level of participants			
Good knowledge			208 (97.7)
Poor knowledge			5 (2.3)

**Table 2:** Socio-demographic characteristics of respondents.

Variable	Frequency (*n* = 213)	Percentage (%)
Age of the child (in months)		
24–36	121	56.8
37–48	65	30.5
49–59	27	12.7

Sex of the child		
Male	110	51.6
Female	103	48.4

Birth weight of child missing	18	8.5
Below 2.5	28	13.1
2.5–4.0	165	77.5
Above 4.0	2	0.9

Age of the caregiver (in completed years)		
20–29	99	46.5
30–39	90	42.2
40–49	20	9.4
**≥50**	4	1.9

Sex of the caregiver		
Male	10	4.7
Female	203	95.3

Caregiver's relationship with the child		
Parent	195	91.5
Other relative	18	8.5

Marital status of the caregiver		
Married	208	97.7
Single	5	2.3

Type of marriage (*n* = 208)		
Monogamous	133	73.2
Polygamous	75	26.8

Occupation of the caregiver		
Government worker	8	3.8
Trader	52	24.4
Farmer	153	

Level of education of the caregiver		
Primary	46	21.6
Junior High School (JHS)	29	13.6
Senior High School (SHS)	20	9.4
Tertiary	7	3.3
None	111	52.1

Religion of the caregiver		
Christian	59	27.7
Islam	149	70.0
African traditional	5	2.3

Tribe of the caregiver		
Kusaasi	126	59.2
Bimoba	29	13.6
Bissa	45	21.1
Mossi	13	6.1

**Table 3:** Child and caregiver factors influencing CWC completion.

Factors	Completed CWC by age		Total (*n* = 213) *n* (%)	Fisher's exact (*p* value)	COR (95% CI) *p* value	AOR (95% CI) *p*-value
	Yes (*n* = 100) *n* (%)	No (*n* = 113) *n* (%)				
Age of the child (months)						
24–36	69 (69.0)	52 (46.0)	121 (56.8)	(0.002)^*∗*^		
37–48	24 (24.0)	41 (36.3)	65 (30.5)		0.44 (0.24–0.82), 0.010	0.42 (0.21–0.86), 0.017
49–59	7 (7.0)	20 (17.7)	27 (12.7)		0.26 (0.10–0.67) 0.005	0.27 (0.10–0.77), 0.014

Marital status of the caregiver						
Single	5 (5.0)	0 (0.0)	5 (2.3)	(0.022)^*∗*^	Ref.	1
Married	95 (45.7)	113 (54.3)	208 (97.7)		1.12 (0.42–2.95), 0.824	
Polygamous	33 (33.0)	47 (41.6)	80 (37.6)		0.69 (0.40–1.21), 0.197	

Occupation of the caregiver						
Gov't worker	8 (100.0)	0 (0.0)	8 (3.8)	(0.003)^*∗*^	Ref.	
Trader	27 (51.9)	25 (48.1)	52 (24.4)		1.30 (0.29–0.97), 0.409	1.05 (0.50–2.21), 0.899
Farmer	65 (42.5)	88 (57.5)	153 (71.8)		0.58 (0.29–0.97), 0.038	1

Caregiver's level of education						
Primary	24 (24.0)	22 (19.5)	46 (21.6)	(*p* ≤ 0.001)^*∗*^	Ref.	
JHS	16 (16.0)	13 (11.5)	29 (13.6)		1.13 (0.44–2.87), 0.800	0.97 (0.35–2.65), 0.945
SHS	17 (17.0)	3 (2.7)	20 (9.4)		5.19 (1.34–20.18), 0.017	4.59 (1.11–19.06), 0.036
Tertiary	7 (7.0)	0 (0.0)	7 (3.3)		1	1
None	36 (36.0)	75 (66.4)	111 (52.1)		0.44 (0.22–0.89), 0.022^*∗*^	0.45 (0.21–0.95), 0.036

Knowledge of the caregiver on CWC						
Poor knowledge	10 (10.0)	27 (23.9)	37 (17.4)	(0.011)^*∗*^	Ref.	
Good knowledge	90 (90.0)	86 (76.1)	176 (82.6)		9.49 (4.59–19.65), *p* ≤ 0.001^*∗*^	0.29 (0.04–2.05), 0.215
Poor	14 (50.0)	14 (50.0)	28 (13.1)		1.15 (0.52–2.55), 0.729	

## Data Availability

The data used to support the findings of this study are available from the corresponding author upon reasonable request.
